# An Unsupervised Classification Algorithm for Heterogeneous Cryo-EM Projection Images Based on Autoencoders

**DOI:** 10.3390/ijms24098380

**Published:** 2023-05-06

**Authors:** Xiangwen Wang, Yonggang Lu, Xianghong Lin, Jianwei Li, Zequn Zhang

**Affiliations:** 1 College of Computer Science and Engineering, Northwest Normal University, Lanzhou 730070, China; wangxw2015@nwnu.edu.cn (X.W.); linxh@nwnu.edu.cn (X.L.); 2021222178@nwnu.edu.cn (Z.Z.); 2 School of Information Science and Engineering, Lanzhou University, Lanzhou 730000, China; 220220942961@lzu.edu.cn

**Keywords:** cryo-electron microscopy, single-particle reconstruction, structural heterogeneity, autoencoder, unsupervised classification

## Abstract

Heterogeneous three-dimensional (3D) reconstruction in single-particle cryo-electron microscopy (cryo-EM) is an important but very challenging technique for recovering the conformational heterogeneity of flexible biological macromolecules such as proteins in different functional states. Heterogeneous projection image classification is a feasible solution to solve the structural heterogeneity problem in single-particle cryo-EM. The majority of heterogeneous projection image classification methods are developed using supervised learning technology or require a large amount of a priori knowledge, such as the orientations or common lines of the projection images, which leads to certain limitations in their practical applications. In this paper, an unsupervised heterogeneous cryo-EM projection image classification algorithm based on autoencoders is proposed, which only needs to know the number of heterogeneous 3D structures in the dataset and does not require any labeling information of the projection images or other a priori knowledge. A simple autoencoder with multi-layer perceptrons trained in iterative mode and a complex autoencoder with residual networks trained in one-pass learning mode are implemented to convert heterogeneous projection images into latent variables. The extracted high-dimensional features are reduced to two dimensions using the uniform manifold approximation and projection dimensionality reduction algorithm, and then clustered using the spectral clustering algorithm. The proposed algorithm is applied to two heterogeneous cryo-EM datasets for heterogeneous 3D reconstruction. Experimental results show that the proposed algorithm can effectively extract category features of heterogeneous projection images and achieve high classification and reconstruction accuracy, indicating that the proposed algorithm is effective for heterogeneous 3D reconstruction in single-particle cryo-EM.

## 1. Introduction

Structural biology is an interdisciplinary field that focuses on the three-dimensional (3D) spatial structure, dynamic processes, and biological functions of biological macromolecules [1]. Its main goal is to study the 3D structure of biological macromolecules and their functions in cells. Cryo-electron microscopy (cryo-EM) is a representative technology in the field of structural biology to resolve the 3D structure of proteins or other biological macromolecules [2,3,4] and is of great importance in the fields of protein design and drug design [5]. It does not require crystallization of biological samples and can resolve 3D structures of biological macromolecules close to their physiological state. The single-particle analysis (SPA) technique, also known as single-particle 3D reconstruction, is one of the fastest growing, most widely used and successful techniques in cryo-EM 3D reconstruction, which is mainly applicable to 3D structure analysis of biological macromolecules with homogeneous structures, and the resolution has reached atomic resolution [6,7,8]. Cryo-EM single-particle 3D reconstruction has been widely used to study the high-resolution 3D structures of biological macromolecular complexes that are difficult to crystallize [9,10]. It is a revolutionary structural biology method for determining the 3D structure of biological macromolecules and has achieved remarkable success in recent years [11,12,13]. As a result of these exciting new developments, cryo-EM was selected as “Method of the Year 2015” by Nature Methods, and the 2017 Nobel Prize in Chemistry was awarded to Jacques Dubochet, Joachim Frank, and Richard Henderson for “developing cryo-electron microscopy (cryo-EM) for the high-resolution structure determination of biomolecules in solution”. In recent years, cryo-EM single-particle 3D reconstruction technologies have been continuously improved and developed [14,15,16].

The structural heterogeneity of flexible biological macromolecules refers to the fact that the two-dimensional (2D) cryo-EM projection images involved in the 3D reconstruction are projected from different 3D structures, which is a prominent research topic in the field of cryo-EM 3D reconstruction and has received widespread attention from researchers [17]. Biological macromolecules are flexible rather than rigid, so their different functional states correspond to different conformations. Since the single-particle cryo-EM technique can directly obtain the 3D structures of biological macromolecules in vitreous ice, it is not necessary to form a highly ordered crystal arrangement of biological macromolecules in the sample, which results in the conformational flexibility of the biological macromolecules themselves not being fixed. This structural heterogeneity problem is called the conformational heterogeneity. In addition, during the sample grid preparation process, if the sample purification is not done well, or samples of some biological macromolecules themselves are difficult to be separated, resulting in a poor purity of the obtained biological samples containing different biological macromolecules. This structural heterogeneity problem is called the compositional heterogeneity. Since the structural heterogeneity of biological macromolecules caused by compositional heterogeneity and conformational heterogeneity is objective and unavoidable, the collected 2D projection images usually do not belong to a single 3D structure, but are a mixture of projection images of several different 3D structures, that is, these 2D projection images are heterogeneous. The methods for reconstructing the 3D structures of flexible biological macromolecules from heterogeneous cryo-EM projection images are called the heterogeneous 3D reconstruction. The different conformations of biological macromolecules reflect their different structural forms when they perform their biological functions, and it is important to analyze and understand the conformational heterogeneity in different functional states and reconstruct the corresponding 3D structures to study the dynamics and principles of biological macromolecules and explain their biological functions [18,19,20]. Heterogeneous 3D reconstruction of flexible biological macromolecules has become a technological difficulty as well as a research hotspot in modern single-particle cryo-EM.

Although single-particle 3D reconstruction methods have been able to resolve the atomic-resolution 3D structures of biological macromolecules, there are still many difficulties in directly reconstructing multiple different 3D structures of the same biological macromolecule in different functional states using this technique. This is because an important prerequisite for single-particle 3D reconstruction techniques is that all 2D projection images involved in the reconstruction are projected from the same 3D structure, that is, all cryo-EM projection images are homogeneous [21,22]. Directly applying traditional single-particle 3D reconstruction methods to process heterogeneous projection images will affect the accuracy and resolution of 3D reconstruction, and fail to reveal important conformational information of flexible biological macromolecules, further limiting the analysis of their biological functions. Therefore, the traditional single-particle 3D reconstruction methods can no longer be directly applied to the problem of structural heterogeneity analysis of flexible biological macromolecules, and heterogeneous 3D reconstruction methods in single-particle cryo-EM need to be explored.

The heterogeneous 3D structures of flexible biological macromolecules are not directly observed, but reconstructed from the corresponding 2D projection images. In order to reconstruct the high-resolution heterogeneous 3D structure of biological macromolecules, all 2D heterogeneous projection images involved in the 3D reconstruction should be divided into several subsets of homogeneous projection images representing 3D structures of different biological macromolecules or different conformations of the same flexible biological macromolecule in different functional states, and then the traditional single-particle 3D reconstruction methods, such as RELION [23] and cryoSPARC [24], can be used directly to reconstruct the corresponding preliminary 3D structures. Therefore, heterogeneous projection image classification is a feasible method to solve the structural heterogeneity problem in single-particle cryo-EM [25,26]. The advantage of such methods is that there is no need to redesign the new 3D reconstruction algorithms, and the existing 3D reconstruction methods can be used directly. However, due to the extremely similar projection images of conformations of many flexible biological macromolecules in different functional states, as well as the very high background noise and different projection orientations in 2D projection images, accurate classification of heterogeneous projection images becomes extremely difficult.

In recent years, researchers have proposed a large number of heterogeneous projection image classification algorithms using different methods to solve the structural heterogeneity problem of biological macromolecules in single-particle cryo-EM [27,28,29]. From the perspective of known 3D reference structure information during classification, the existing algorithms for heterogeneous projection image classification can be divided into two categories, that is, supervised classification algorithms based on the known 3D template structures and unsupervised classification algorithms without any a priori knowledge of heterogeneous 3D template structures.

In the supervised classification algorithms, heterogeneous projection images are classified into different subsets of homogeneous projection images using a priori knowledge of heterogeneous 3D structures and conformational states of flexible biological macromolecules, where the critical process is projection matching. In other words, this kind of methods use known 3D structures as template structures to achieve heterogeneous projection image classification according to the similarity between cryo-EM heterogeneous projection images and synthetic projection images projected from the 3D template structures [30,31]. It can be seen that accurate prior knowledge of heterogeneous 3D structures of flexible biological macromolecules is the key to such methods. The advantage of supervised classification algorithms is that the classification accuracy of heterogeneous projection images is relatively high. The main drawback is that they rely too much on a priori knowledge of heterogeneous 3D structures and conformational states of flexible biological macromolecules. If there are no 3D template structures or the 3D template structures are inaccurate, the corresponding heterogeneous 3D structures and conformational states, alignment parameters, and projection orientations of the heterogeneous projection images assigned during the projection matching process will also be inaccurate, resulting in inaccurate reconstruction of the heterogeneous 3D structures. Therefore, supervised classification algorithms have significant limitations in practical applications.

In the unsupervised classification algorithms, heterogeneous projection images are classified into different subsets of homogeneous projection images based on traditional clustering algorithms according to the clustering characteristics of heterogeneous projection images. This kind of method calculates the distance metric between heterogeneous projection images based on the critical features of projection images such as the common lines between projection images and the class averages corresponding to the original projection images, and uses clustering algorithms combined with principal component analysis and maximum likelihood expectation maximization to achieve heterogeneous projection image classification. We proposed a weighted voting algorithm [14] that can calculate the similarity and reliability of common lines and apply it to the unsupervised classification of heterogeneous projection images, and proposed an unsupervised classification algorithm [32] for heterogeneous projection images based on the similarity and reliability of common lines, as well as a two-stage unsupervised classification algorithm [33] that combines common lines and class averages. Wu et al. [34] proposed an unsupervised classification algorithm for projection images based on statistical manifold learning. Pothula et al. [35] applied the K-means algorithm to classify projection images of helical protein polymers. Verbeke et al. [36] proposed a projection image classification algorithm based on common lines and graph clustering. Gomez-Blanco et al. [37] proposed a projection image classification algorithm based on hierarchical clustering. The advantage of unsupervised classification algorithms is that they usually only need to know the number of heterogeneous 3D structures of flexible biological macromolecules in the heterogeneous cryo-EM projection image dataset, without a priori knowing the specific structural information of each heterogeneous 3D structure and conformational state, and thus have a wider application than the supervised classification algorithms. The main drawback is that it is very difficult to accurately compute the critical features of the projection images, such as common lines, because the very low electron dose during sample preparation results in very high background noise in the generated projection images [38,39]. In addition, the common lines of the projection images are usually computed in Fourier space based on the similarity between the radial lines of all projection images, which is very time-consuming [21,22]. For example, to compute the common line between the projection images *i* and *j*, their polar Fourier transforms are usually discretized into 360 radial lines, and then the similarity between each pair of radial lines is computed to find the most similar pair of radial lines, which is the common line between the projection images *i* and *j*.

With the rapid development of machine learning methods, deep learning has become a breakthrough technique in the field of advanced machine learning, making great breakthroughs in many areas of pattern recognition. Deep learning models have strong data representation and feature learning capabilities, and have unique advantages in studying heterogeneous 3D reconstruction problems in single-particle cryo-EM [40,41,42,43,44]. Recently, some heterogeneous 3D reconstruction algorithms in single-particle cryo-EM based on neural network and deep learning techniques have been proposed [45,46,47,48,49,50], including supervised and unsupervised classification algorithms. In addition, we have proposed an unsupervised classification algorithm for heterogeneous projection images based on deep autoencoders [51]. This kind of methods uses deep generative neural network models such as autoencoders implemented with feedforward networks to learn the potential knowledge of heterogeneous 3D structures from the heterogeneous projection images, encode the heterogeneous projection images into latent space, and analyze the heterogeneous 3D structures and conformational states of flexible biological macromolecules according to the distribution of latent variables in the latent space. An important prerequisite of such methods is that they require a priori knowledge such as the projection orientations of the heterogeneous projection images. The accuracy of cryo-EM heterogeneous 3D reconstruction is directly determined by the accuracy of the estimated projection orientations of the heterogeneous projection images. However, it is difficult to accurately estimate their orientations because the signal-to-noise ratio of the heterogeneous projection images is very low, which leads to certain limitations of these deep learning-based algorithms in practical applications.

Autoencoder is one of the most commonly used generative neural network models that has been extensively applied to many unsupervised learning scenarios. Autoencoder models achieve feature representation learning of the data through consistency between the input of the encoder network and the output of the decoder network. The core task of heterogeneous projection image classification is to compute the category features that can be used to distinguish which 3D structure the heterogeneous projection images belong to. In this paper, an unsupervised heterogeneous projection image classification algorithm based on autoencoders is proposed, which can effectively classify heterogeneous projection images into homogeneous subsets without any a priori knowledge other than the number of heterogeneous 3D structures. Specifically, (1) a simple autoencoder model with multi-layer perceptrons and a complex autoencoder model with residual networks are implemented to extract the category features of heterogeneous projection images. For the convenience of description, the autoencoder model implemented by multi-layer perceptrons will be referred to as AE-MLP and the autoencoder model implemented by residual networks will be referred to as AE-RES in the remainder of this paper. The AE-MLP model needs to be trained in the iterative mode, while the AE-RES model can be trained in the one-pass learning mode [52,53]. (2) The high-dimensional features extracted by the autoencoders are reduced to 2D using the uniform manifold approximate and projection (UMAP) algorithm [54], which is a commonly used dimensionality reduction method in life sciences [55,56]. (3) These 2D features are clustered by the spectral clustering algorithm [57,58] and thus the unsupervised classification of heterogeneous projection images is achieved. After classifying the heterogeneous projection images into homogeneous projection image subsets, the traditional single-particle 3D reconstruction methods are used to separately reconstruct the corresponding preliminary 3D volume in each classified homogeneous projection image subset.

Compared to other heterogeneous projection image classification algorithms [31,33,45], the proposed unsupervised heterogeneous projection image classification algorithm based on autoencoders has the following advantages:

- Compared to supervised heterogeneous projection image classification algorithms [30,31], the proposed algorithm does not require 3D template structures of heterogeneous conformations, so its application is broader.
- Compared to traditional unsupervised heterogeneous projection image classification algorithms based on common lines [32,33], the proposed algorithm does not require a priori knowledge such as the common lines of projection images, so its computation is faster.
- Compared to the heterogeneous projection image classification algorithms based on deep learning [45,46,47], the proposed algorithm does not require a priori knowledge such as the orientations of the projection images, so its robustness is stronger.

The main contributions of this paper can be summarized as follows:

- We propose an unsupervised classification algorithm for heterogeneous projection images based on autoencoders, which relies only on the information of the heterogeneous projection images themselves and does not require any other prior knowledge such as the projection orientations.
- We implement a multi-layer perceptron-based autoencoder and a residual network-based autoencoder to transform heterogeneous projection images into latent variables, which can automatically extract the category features of heterogeneous projection images.
- Compared to other heterogeneous projection image classification algorithms, the proposed algorithm has a broader application scope, faster computation speed, and stronger robustness.

The remainder of this paper is organized as follows. In Section 2, the classification performance of the proposed algorithm is demonstrated on two synthetic datasets composed of heterogeneous cryo-EM projection images. The results of the heterogeneous 3D reconstruction are also presented. In Section 3, the proposed unsupervised autoencoder-based heterogeneous projection image classification algorithm is introduced in detail, including the general framework of heterogeneous 3D reconstruction in single-particle cryo-EM and two specific autoencoder models. Finally, the paper is concluded in Section 4.

## 2. Results and Discussion

### 2.1. Experimental Setup

In the experiments, the performance of the proposed unsupervised heterogeneous projection image classification algorithm based on autoencoders is demonstrated on synthetic heterogeneous cryo-EM projection image datasets. The source of structural heterogeneity of biological macromolecules in cryo-EM images is well known and broadly attributed due to compositional during sample grid preparation and conformational heterogeneity corresponding to different states of the biological macromolecules. Therefore, we have prepared two datasets, one is the discrete compositional heterogeneous conformational dataset composed of different biological macromolecules, and the other one is the conformational heterogeneous dataset composed of different conformations of the same flexible biological macromolecule in different functional states. The heterogeneous projection images are projected from different published 3D density maps and their orientations are uniformly distributed over the rotation group SO(3). The real cryo-EM projection images contain a high level of background noise with a signal-to-noise ratio of about 0.1. In order to make the synthetic noise projection images close to the real cryo-EM projection image, as in many studies that generate synthetic noisy projection images [21,22], the Gaussian white noise with the fixed signal-to-noise ratio SNR=0.1 is added to the synthetic heterogeneous cryo-EM projection images. The signal-to-noise ratio is defined as follows:

(1) $$SNR=\frac{\mathrm{var}(signal)}{\mathrm{var}(noise)}$$

where var is the variance (energy), signal is the clean projection image, and noise is the noise realization of this projection image.

The proposed AE-MLP and AE-RES models are implemented in the PyTorch software and are trained using the stochastic gradient descent (SGD) algorithm in a minibatch manner. Table 1 lists the main parameters used for training the AE-MLP and AE-RES models. The classification results of the proposed AE-MLP and AE-RES models are compared with the clustering results of the original heterogeneous projection images to further demonstrate their classification effectiveness.

All synthetic heterogeneous cryo-EM projection images are downsampled to images with a size of 64×64. For the AE-RES model, the single-channel projection images are converted to three-channel images by twice replications. The pre-processed projection images are input into the autoencoder model for feature extraction and the UMAP dimensionality reduction algorithm is used to reduce the dimension of the extracted high-dimensional features to 2D. The normalized spectral clustering algorithm is used to classify heterogeneous projection images into homogeneous groups according to the obtained 2D features. The classification accuracy of heterogeneous projection images, defined as the ratio of the number of correctly classified projection images to the total number of projection images, is used to measure the classification performance of the proposed unsupervised classification algorithm.

After dividing the heterogeneous cryo-EM projection images into homogeneous groups, the projection images in each group are used separately to reconstruct a preliminary 3D density map. The heterogeneous projection images are downsampled for classification, while the original, undownsampled projection images are used for 3D reconstruction. The Fourier-based 3D reconstruction package FIRM [59] embedded in the ASPIRE software package is used for reconstruction from the classified projection images with the corresponding ground truth projection orientations. The 3D Fourier Shell Correlation (FSC) [60], which measures the normalized cross-correlation coefficient between two 3D density maps over corresponding spherical shells in Fourier space, is used to evaluate the accuracy of the reconstructions. All cryo-EM 3D density maps are visualized using the UCSF ChimeraX software [61,62].

### 2.2. Classification of Discrete Compositional Conformations

**Figure 1:**
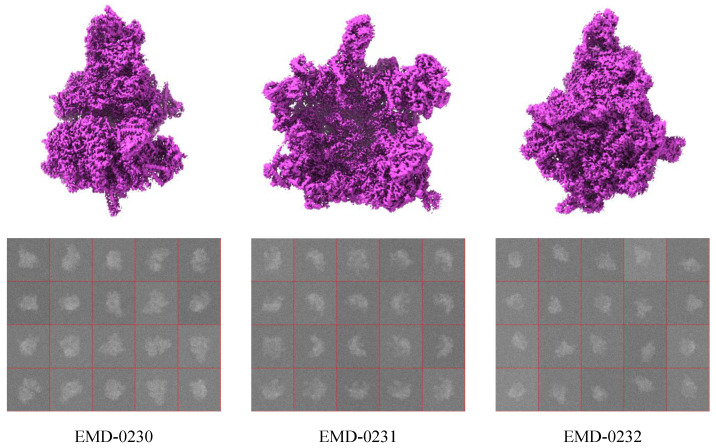
The discrete compositional conformation cryo-EM dataset. Top row: the published 3D density maps EMD-0230, EMD-0231, and EMD-0232. Bottom row: some synthetic noisy projection images belonging to these three 3D density maps.

In this experiment, the classification performance of the AE-MLP and AE-RES models is validated on a relatively simple discrete compositional conformation cryo-EM dataset. The dataset contains synthetic projection images projected from three different ribosomes, namely the Trypanosoma brucei mitochondrial ribosome small subunit in complex with mt-IF3 (EMD-0230), the Trypanosoma brucei mitochondrial ribosome large subunit (EMD-0231), and the Trypanosoma brucei mitochondrial ribosome small subunit body in complex with mt-IF3 (EMD-0232) [63]. The reported resolutions of these three different ribosomes are 3.37 Å, 3.39 Å, and 3.27 Å, respectively. The voxel dimensions and the map dimensions of these ribosomes are 1.39×1.39×1.39 Å and 320×320×320 voxels, respectively. We use each 3D density map to generate 500 noisy projection images with a size of 320×320. In this way, the first heterogeneous cryo-EM projection image dataset contains 1500 noisy projection images belonging to the three 3D structures. Figure 1 shows the three published cryo-EM 3D structures and some synthetic noisy projection images belonging to these three 3D density maps in this dataset.

**Figure 2:**
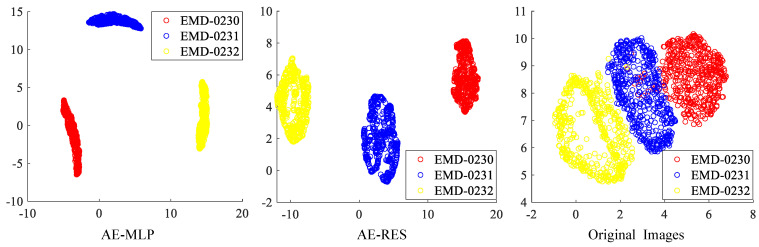
Visualization of the 2D features acquired from the AE-MLP model, the AE-RES model and the original heterogeneous projection images in the discrete compositional conformation cryo-EM dataset using the UMAP dimensionality reduction algorithm.

After preprocessing these heterogeneous projection images, they are input into the AE-MLP and AE-RES models respectively to extract their category features. The heterogeneous projection images are encoded as 8D and 4096D latent variables respectively after training the AE-MLP model and the AE-RES model. The latent variables and the original heterogeneous projection images are reduced to 2D by using the UMAP dimensionality reduction algorithm. Figure 2 illustrates the visualization results of these 2D features. It shows that the features of the heterogeneous projection images belonging to these three density maps extracted by the AE-MLP model and the AE-RES model are both independently distributed in 2D space, indicating that both the AE-MLP model and the AE-RES model can effectively extract the category features of the heterogeneous projection images. The original heterogeneous projection images after direct dimensionality reduction are not completely independently distributed in 2D space, which further illustrates the effectiveness of the proposed algorithm in feature extraction.

Table 2 lists the classification accuracy on the discrete compositional conformation cryo-EM dataset using the 2D features acquired from the AE-MLP model, the AE-RES model, and the original heterogeneous projection images. It can be seen that using the 2D features extracted by both the AE-MLP model and the AE-RES model can accurately classify the heterogeneous projection images belonging to the three density maps, while the overall classification accuracy of directly using the original heterogeneous projection images is 96.00%.

### 2.3. Classification of Conformations of the Bacterial Ribosome

**Figure 3:**
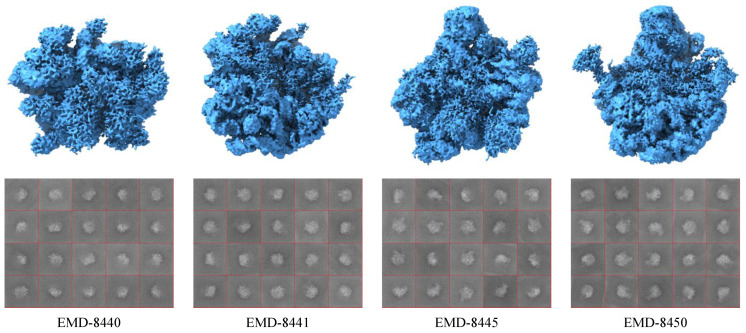
The conformations of the bacterial ribosome cryo-EM dataset. Top row: the published 3D density maps EMD-8440, EMD-8441, EMD-8445, and EMD-8450. Bottom row: some synthetic noisy projection images belonging to these four 3D density maps.

In this experiment, the classification performance of the AE-MLP and AE-RES models is validated on a highly heterogeneous cryo-EM dataset containing synthetic projection images projected from four conformations of the bL17-depleted large ribosomal subunit assembly intermediate (EMD-8440, EMD-8441, EMD-8445, and EMD-8450) [64]. The reported resolutions of these four conformations are 4.5 Å, 3.7 Å, 4.0 Å, and 3.7 Å, respectively. The voxel dimensions and the map dimensions of them are 1.31×1.31×1.31 Å and 320×320×320 voxels, respectively. We use each 3D density map to generate 500 noisy projection images with a size of 320×320. In this way, the second heterogeneous cryo-EM projection image dataset contains 2000 noisy projection images belonging to the four conformations. Figure 3 shows the four published cryo-EM 3D structures that were reconstructed from the cryo-EM dataset of L17-depleted 50S ribosomal intermediates (EMPIAR-10076) [64] and some synthetic noisy projection images belonging to these four 3D density maps in this dataset.

**Figure 4:**
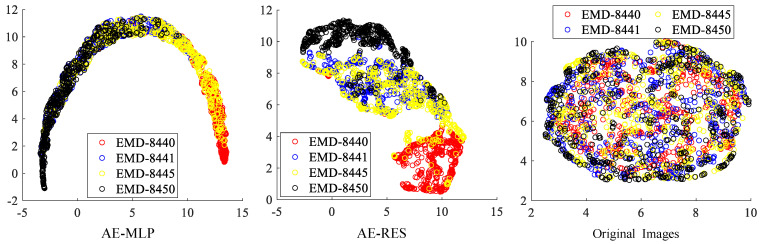
Visualization of the 2D features acquired from the AE-MLP model, the AE-RES model and the original heterogeneous projection images in the conformations of the bacterial ribosome cryo-EM dataset after dimensionality reduction using the UMAP algorithm.

The category features of heterogeneous projection images belonging to the four conformations of the bL17-depleted large ribosomal subunit assembly intermediate are extracted by the AE-MLP model and the AE-RES model. The high-dimensional latent variables and the original heterogeneous projection images are reduced to 2D after dimensionality reduction using the UMAP algorithm. The visualization results of these 2D features are illustrated in Figure 4. It can be seen that, compared to the simple dataset in the first experiment, the features extracted using the AE-MLP model and the AE-RES model on the complex dataset in this experiment are not as independently distributed as in the first experiment, but they still show more aggregation characteristics than directly using the original heterogeneous projection images.

The spectral clustering accuracy of the 2D features extracted by different models are listed in Table 3. It can be seen that the AE-RES model has the highest classification accuracy with 67.25%, followed by the AE-MLP model with 57.60%, and the direct use of original heterogeneous projection images has the lowest classification accuracy with 26.90%. This experiment further illustrates that the proposed algorithm is effective in the unsupervised classification of heterogeneous projection images.

In general, both the AE-MLP model and the AE-RES model can effectively extract the category features of heterogeneous projection images. The first dataset is a relatively simple dataset of projection images belonging to discrete compositional conformations, and both the AE-MLP model and the AE-RES model can be applied to accurately classify heterogeneous projection images. The second dataset contains projection images belonging to four conformations of the bL17-depleted large ribosomal subunit assembly intermediate, which is very difficult to classify. The classification accuracy of the AE-MLP model and the AE-RES model on the second dataset is much higher than that of directly using the original projection images. The AE-MLP model requires iterations, but runs very fast due to its simple structure. The AE-RES model has a complex structure, but its learning and representation capabilities are so strong that the desired results can be obtained even in the one-pass learning mode.

### 2.4. Heterogeneous Cryo-EM 3D Reconstruction of the Bacterial Ribosome

In this experiment, we reconstruct the preliminary 3D density maps of corresponding biological macromolecules based on the classification results of heterogeneous projection images to achieve heterogeneous cryo-EM 3D reconstruction. The results of heterogeneous cryo-EM 3D reconstruction are mainly affected by the accuracy of heterogeneous projection image classification, the number of projection images involved in the 3D reconstruction, and the projection orientations of the projection images. The main purpose of this paper is to propose an efficient heterogeneous projection image classification algorithm, so we use the ground-truth projection orientations of the projection images to reconstruct the preliminary 3D density maps. In this way, the reconstruction accuracy is only related to the classification accuracy of the projection images and the number of projection images involved in the reconstruction. The higher the classification accuracy of the projection images and the more projection images involved in the reconstruction, the higher the reconstruction accuracy. The heterogeneous projection images on the discrete compositional conformation dataset in Section 2.2 are almost accurately classified, so we only implement heterogeneous cryo-EM 3D reconstruction on the bacterial ribosome dataset in Section 2.3.

Table 4 lists the number of projection images in each group on the conformations of the bacterial ribosome cryo-EM dataset using the 2D features acquired from the AE-MLP model, the AE-RES model, and the original heterogeneous projection images. The projection images classified to the same group will be used to reconstruct the corresponding preliminary 3D density map.

**Figure 5:**
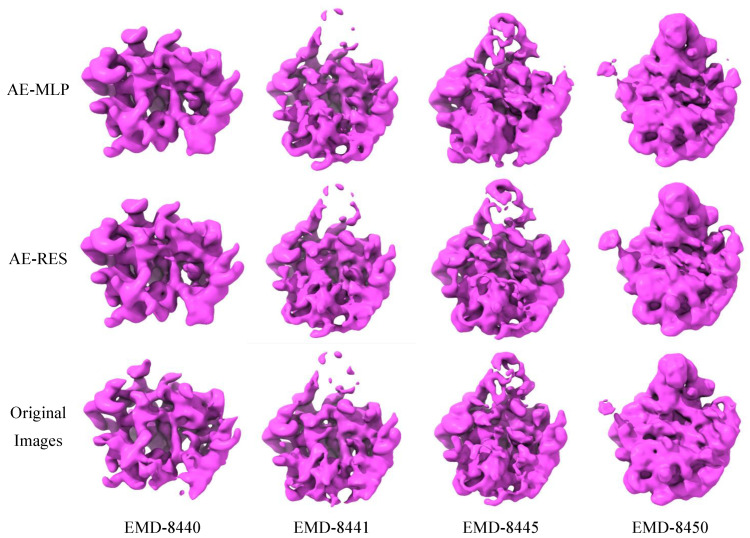
The preliminary 3D density maps of EMD-8440, EMD-8441, EMD-8445, and EMD-8450 reconstructed from the classification results of the 2D features acquired from the AE-MLP model, the AE-RES model, and the original heterogeneous projection images.

Figure 5 shows the preliminary 3D density maps of EMD-8440, EMD-8441, EMD-8445, and EMD-8450 reconstructed from the classification results of the 2D features acquired from the AE-MLP model, the AE-RES model, and the original heterogeneous projection images. It can be seen that the classification results based on the 2D features acquired from the AE-MLP model, the AE-RES model and the original image are all able to reconstruct the corresponding preliminary 3D density maps, but the preliminary 3D density maps reconstructed based on the classification results of the 2D features acquired from the AE-RES model are more similar to the published 3D density maps shown in the top row of Figure 3.

**Figure 6:**
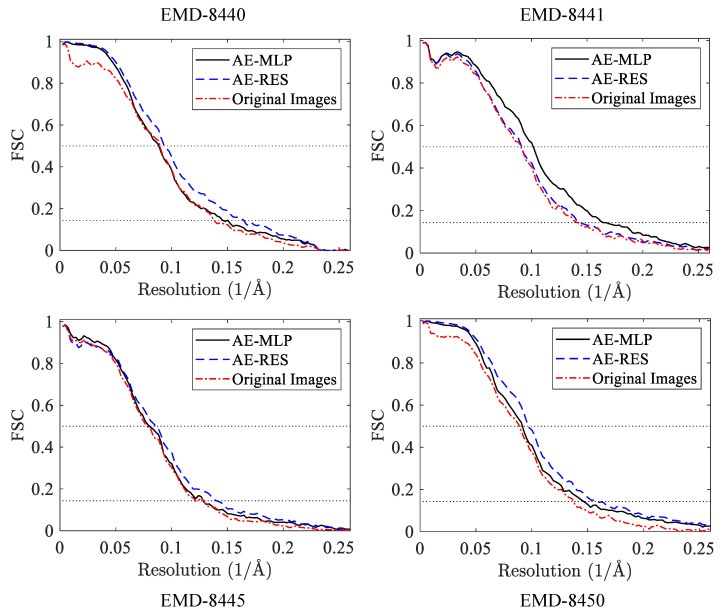
FSC curves of the preliminary 3D density maps of EMD-8440, EMD-8441, EMD-8445, and EMD-8450 reconstructed from the classification results of the 2D features acquired from the AE-MLP model, the AE-RES model, and the original heterogeneous projection images.

Figure 6 shows the FSC curves of the preliminary 3D density maps of EMD-8440, EMD-8441, EMD-8445, and EMD-8450 reconstructed from the classification results of the 2D features acquired from the AE-MLP model, the AE-RES model, and the original heterogeneous projection images. These FSC curves are calculated against the corresponding published 3D density maps shown in Figure 3. It can be seen that using the classification results of the 2D features acquired from the AE-RES model can achieve higher reconstruction accuracy than using the classification results of the 2D features acquired from the AE-MLP model and the original heterogeneous projection images. This is because the classification accuracy of the heterogeneous projection images using the 2D features acquired from the AE-RES model is higher than that of using the 2D features acquired from the AE-MLP model and the original heterogeneous projection images. For EMD-8441, the highest reconstruction accuracy was achieved using the AE-MLP model, which is due to the fact that the classification accuracy of the heterogeneous projection images using the AE-MLP model is 60.40%, which is higher than the classification accuracy of the heterogeneous projection images using the AE-MLP model and the original projection images. In addition, the number of projection images involved in the reconstruction after classification by the AE-MLP model is 676, which is much more than the number of projection images involved in the reconstruction after classification by the AE-RES model and the original projection images. The results of heterogeneous 3D reconstruction show that high classification accuracy of heterogeneous projection images can lead to high 3D reconstruction accuracy, which further indicates that the proposed autoencoder-based unsupervised classification algorithm for heterogeneous cryo-EM projection images is effective in heterogeneous cryo-EM 3D reconstruction.

## 3. Materials and Methods

In this section, the general framework of heterogeneous 3D reconstruction in single-particle cryo-EM is first presented, where the autoencoder model is used to automatically learn the category features of heterogeneous cryo-EM projection images. Furthermore, the implementation details of two specific autoencoder models are presented, including the AE-MLP model, a simple autoencoder model implemented by multi-layer perceptrons, and the AE-RES model, a complex autoencoder model implemented by deep residual networks.

### 3.1. General Framework of Heterogeneous 3D Reconstruction in Cryo-EM

Autoencoder is one of the most commonly used deep generative network models for feature extraction, which achieves the latent variable representation of data by the consistency between the input of the encoder network and the output of the decoder network in a self-supervised manner. The input data are encoded as latent variables in a latent space after training the autoencoder model, which represent the learned features of the input data. Here, we present a new heterogeneous 3D reconstruction algorithm in single-particle cryo-EM, where an autoencoder model is first used to automatically learn the category features of heterogeneous projection images, and then the extracted high-dimensional features are reduced to 2D features using the UMAP algorithm, and finally these 2D features are used to classify heterogeneous projection images using the spectral clustering algorithm. After classifying the heterogeneous projection images into homogeneous subsets, the corresponding preliminary cryo-EM 3D volume in each homogeneous subset can be reconstructed by using the traditional single-particle 3D reconstruction methods, thus realizing the heterogeneous 3D reconstruction in single-particle cryo-EM.

**Figure 7:**
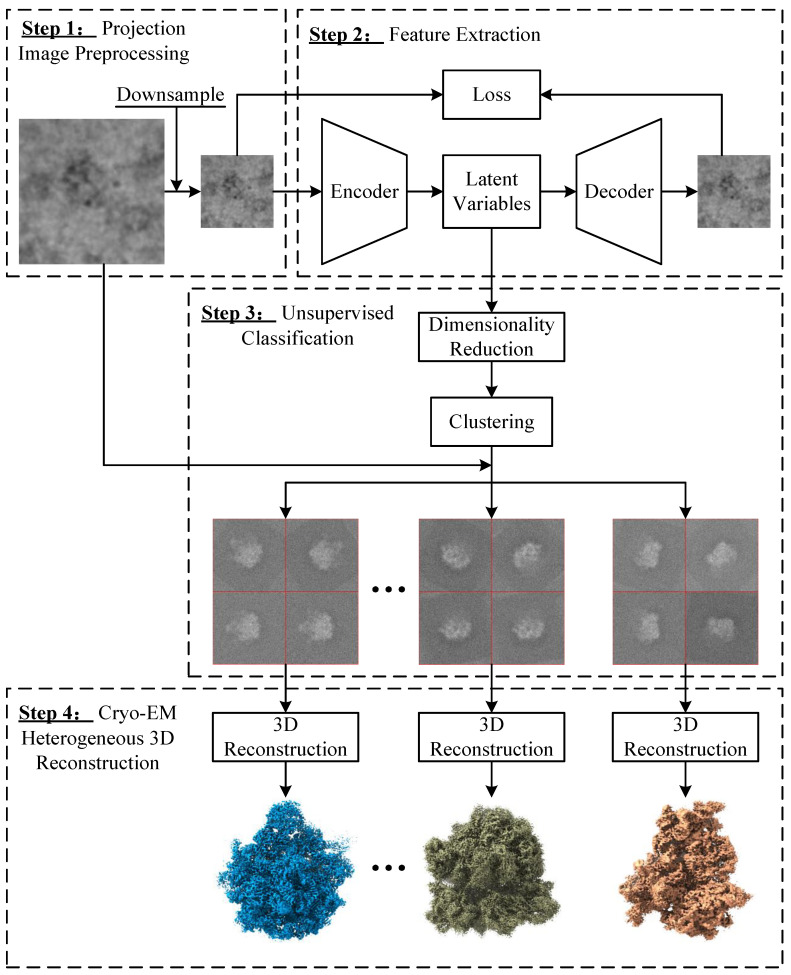
General framework of the proposed heterogeneous 3D reconstruction algorithm based on autoencoders in single-particle cryo-EM, which consists of the following four key steps. Step 1: Projection image preprocessing including image downsampling and increasing the number of channels of the image. Step 2: Feature extraction using the autoencoder model with an encoder-decoder architecture. Step 3: Unsupervised classification of heterogeneous projection images using a normalized spectral clustering algorithm. Step 4: Cryo-EM heterogeneous 3D reconstruction using traditional single-particle 3D reconstruction methods.

Figure 7 illustrates the general framework of the proposed heterogeneous 3D reconstruction algorithm based on autoencoders in single-particle cryo-EM. The proposed algorithm includes the following four main steps:

- Step 1: Projection Image Preprocessing. To take advantage of the powerful image feature learning capability of the autoencoder model, the input projection image needs to undergo some preprocessing operations, including image downsampling and increasing the number of channels of the image. Image downsampling, that is, reducing the size of an image, is one of the basic operations in image processing, which can not only reduce the computational load of subsequent image processing algorithms, but also achieve preliminary denoising of images. In the proposed algorithm, the original heterogeneous projection images with a size of n×n(n>64) are downsampled to a size of 64×64. Moreover, increasing the number of channels of an image can effectively enhance its semantic information, which is also a fundamental operation in computer vision applications based on convolutional neural networks. For the AE-MLP model, the projection images still retain a single channel, while for the AE-RES model, the single-channel projection images are converted to three-channel images by twice replications.
- Step 2: Feature Extraction. The autoencoder model consists of an encoder network and a decoder network. The encoder network transforms the pre-processed projection images into latent variables in a latent space, while the decoder network converts these latent variables to generate images of the same size as the input images. A loss function is then computed based on the input and output images of the autoencoder model, and the error backpropagation algorithm is applied to train the autoencoder model to make the input and output of the autoencoder model as similar as possible by adjusting the network weights. After training the autoencoder model, the representation learning of heterogeneous projection images is achieved, and the category features of heterogeneous projection images are embedded into these latent variables of the autoencoder model.
- Step 3: Unsupervised Classification. This is a crucial step to achieve unsupervised classification of heterogeneous projection images, which consists of the following three key steps.
  − Step 3.1: Feature Dimensionality Reduction. In order to accommodate the performance of the autoencoder model, the dimensionality of the latent variables is usually greater than 2. Therefore, we use the UMAP dimensionality reduction algorithm [54] to reduce the dimensionality of the obtained latent variables to 2D, which can reduce redundant features and further aggregate the most important features, while providing convenience for visualizing the distribution of heterogeneous projection images in the feature space.
  − Step 3.2: Spectral Clustering. The spectral clustering algorithm [57,58] is one of the most commonly used high-performance clustering algorithms in pattern recognition. Therefore, we apply a normalized spectral clustering algorithm [65] to cluster the obtained 2D features. First, the Euclidean distance between these 2D features is computed as a distance matrix *D*, which is further converted into an adjacency matrix $M_{ADJ}$ using a k-nearest neighbor (KNN) algorithm [66] and a shared nearest neighbor (SNN) algorithm [67]. Specifically, the matrix $M_{SNN}$, which represents the number of shared nearest neighbors between 2D features of projection images *i* and *j*, is calculated as follows:
    (2) $$M_{SNN}\left(i,j\right)=\left|M_{KNN}\left(i\right)\cap M_{KNN}\left(j\right)\right|$$
    where $M_{KNN}\left(i\right)$ and $M_{KNN}\left(j\right)$ are the sets of *k* nearest neighbors of the 2D features of the projection images *i* and *j*, respectively, which can be found by the distance matrix *D*. The adjacency matrix $M_{ADJ}$ is converted from the matrix $M_{SNN}$:
    (3) $$M_{ADJ}\left(i,j\right)=\left\{\begin{array}{cc} 1, & M_{SNN}\left(i,j\right)>N_{S}\\ 0, & \mathrm{otherwise} \end{array}\right.$$
    where $N_{S}=5$ is the threshold parameter used to represent at least $N_{S}$ shared nearest neighbors between projection images *i* and *j*. The empirical value of the parameter *k* in the KNN algorithm can be calculated adaptively according to the total number of projection images *N*:
    (4) $$k=\sqrt{N}+N_{S}$$
  − Step 3.3: Unsupervised Classification of Heterogeneous Projection Images. The adjacency matrix $M_{ADJ}$ is used as the input of the normalized spectral clustering algorithm [65] to perform the unsupervised classification of heterogeneous projection images. Finally, the heterogeneous projection images are classified into homogeneous subsets according to the clustering results.
- Step 4: Heterogeneous 3D Reconstruction in Single-Particle Cryo-EM. After classifying the heterogeneous projection images into homogeneous projection image subsets, traditional single-particle 3D reconstruction methods such as RELION [23] and cryoSPARC [24] can be used to separately reconstruct the corresponding initial 3D structure in each classified homogeneous subset.

### 3.2. AE-MLP: Autoencoder Model Implemented by Multi-Layer Perceptrons

**Figure 8:**
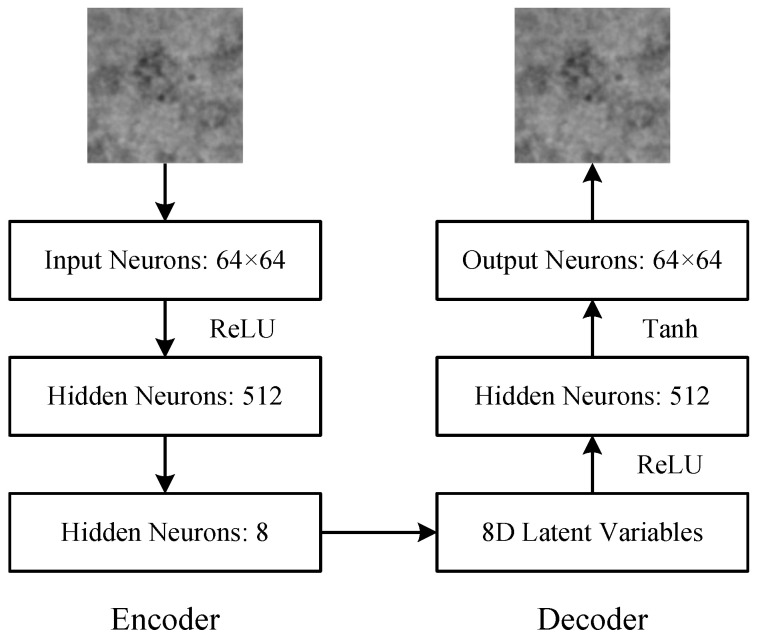
Architecture of the AE-MLP model, which consists of an encoder network and a decoder network. The encoder network is a three-layer fully connected feedforward network with 64×64-512-8 neurons. The decoder network is also a three-layer fully connected feedforward network with 8-512-64×64 neurons. ReLU, rectified linear unit.

The multi-layer perceptron is a classical multi-layer feed-forward fully connected neural network model with a simple network architecture and very powerful feature learning and representation capabilities. The multi-layer perceptron model is used to implement a relatively simple autoencoder model, AE-MLP. Figure 8 illustrates the architecture of the AE-MLP model, which consists of an encoder network and a decoder network. The structure of the encoder and decoder networks in the AE-MLP model is symmetric. The dimensionality of the latent variables in the AE-MLP model is 8. Both the encoder and decoder networks consist of a three-layer fully connected feedforward network.

In the encoding process, the single-channel heterogeneous projection images with a size of n×n(n>64) are first downsampled to images with a size of 64×64 and are input into the encoder network. Then, the downsampled projection images are calculated through the rectified linear unit (ReLU) activation function and are input to a hidden layer containing 512 neurons. Finally, the outputs of this hidden layer are converted into 8D latent variables in the latent space through a linear transformation. The calculation process of the ReLU activation function is as follows:

(5) $$ReLU\left(x\right)=\max\left(0,w^{T}x+b\right)$$

where *w* and *b* denote the weights and bias values of the network, respectively.

In the decoding process, the 8D latent variables are first calculated by the ReLU activation function. Then, the calculation results are input to a hidden layer containing 512 neurons. Finally, the outputs of this hidden layer are decoded as output images with a size of 64×64 through the Tanh activation function. The calculation process of the Tanh activation function is as follows:

(6) $$Tanh\left(x\right)=\frac{e^{x}-e^{-x}}{e^{x}+e^{-x}}$$

During the training process of the AE-MLP model, the network weights are updated to make the inputs and outputs of the AE-MLP model as similar as possible. Specifically, a loss function is first calculated based on the inputs and outputs of the AE-MLP model. The loss function used in the AE-MLP model in this paper is the Mean Squared Error (MSE) function, which is expressed as follows:

(7) $$L=\frac{1}{N}\sum_{i=1}^{N}\left(y_{i}-{\hat{y}}_{i}\right)$$

where $y_{i}$ and ${\hat{y}}_{i}$ denote the input image at the encoder side and the output image at the decoder side, respectively. Then, the error backpropagation algorithm is applied to train the autoencoder model to make the input and output of the autoencoder model as similar as possible by adjusting the network weights. That is, the autoencoder model is trained to make the value of its loss function *L* as small as possible.

The structure of the AE-MLP model is very simple, and the time required for one iteration during the training process is very short. However, in order to extract effective category features from the input heterogeneous projection images, the training of the AE-MLP model requires a significant amount of iterative computation. Therefore, we adopt an iterative training mode to train the AE-MLP model.

### 3.3. AE-RES: Autoencoder Model Implemented by Residual Networks

**Figure 9:**
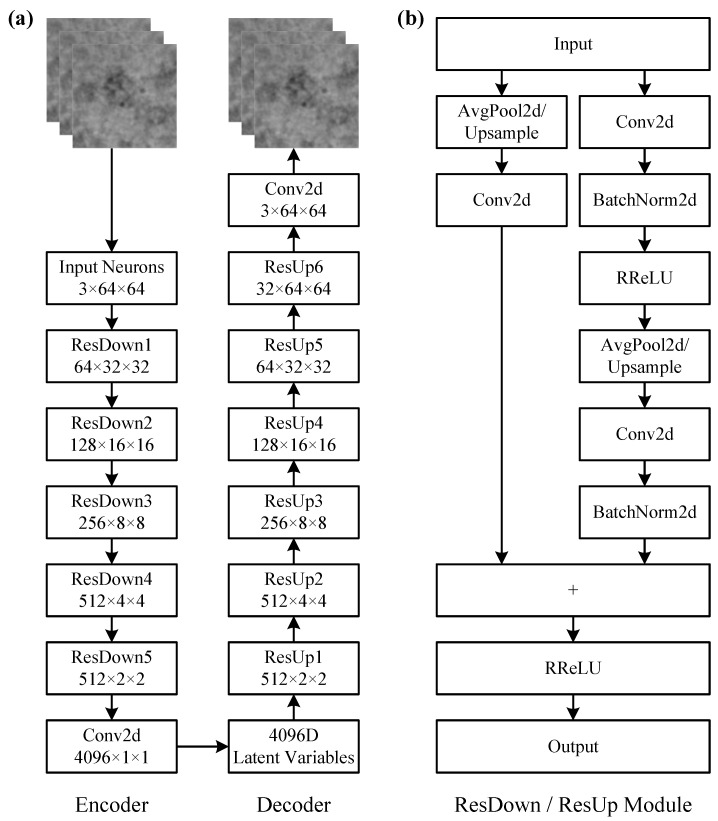
Architecture of the AE-RES model. (**a**) The AE-RES model consists of an encoder network and a decoder network, where the encoder network includes an input layer, five residual downsampling modules ResDown, and a convolution layer, while the decoder network includes six residual upsampling modules ResUp and a convolution layer. (**b**) Architecture of the ResDown and ResUp modules. The ResDown and ResUp modules have the same structure and calculation process, the difference being that the ResDown module uses the 2D average pooling operation AvgPool2d, while the ResUp module uses the upsampling operation Upsample. ResDown, residual downsampling; ResUp, residual upsampling; Conv2d, 2D convolution operation; AvgPool2d, 2D average pooling operation; Upsample, upsampling operation; BatchNorm2d, 2D batch normalization operation; RReLU, randomized leaky rectified linear unit; +, concatenation operation.

Deep residual networks are very important deep learning models that use residual blocks with skip connections to construct the model architecture. They can effectively alleviate some problems in traditional deep learning models, such as gradient disappearance, gradient explosion, and model degradation [68]. Meanwhile, deep residual networks can extract rich high-level features from the input data and effectively improve the feature learning and representation capabilities of deep neural networks, and have the advantages of easy optimization and fast convergence. In addition, deep convolutional neural networks are very powerful and popular deep learning models, especially in the field of computer vision [69]. They have unique advantages in image data processing. Therefore, we use residual networks and convolutional neural networks to implement an autoencoder model AE-RES, which is more complex than the AE-MLP model. Figure 9 illustrates the architecture of the AE-RES model. The dimensionality of the latent variables in the AE-RES model is 4096.

The architecture of the encoder and decoder networks in the AE-RES model is asymmetric. The encoder network consists of an input layer, five residual downsampling modules ResDown, and a convolution layer, while the decoder network consists of six residual upsampling modules ResUp and a convolution layer. The ResDown module is mainly used to transform the input high-dimensional heterogeneous projection images into low-dimensional latent variables in the latent space, while the ResUp module is mainly used to generate the output projection images that consistent with the input projection images from these low-dimensional latent variables.

In the AE-RES model, the heterogeneous projection images undergo a series of complex network computations to extract their category features. The input single-channel projection images are first downsampled to images with a size of 64×64 and are converted to three-channel images by twice replications. Then the three-channel projection images are input into the encoder network and encoded into 4096D latent variables after the computation of the ResDown modules and the convolutional layer. Finally, these 4096D latent variables are decoded as three-channel images with a size of 64×64 in the decoder network after the computation of the ResUp modules and the convolutional layer. The loss function used in the AE-RES model is also the MSE function that expressed in Equation (7).

The ResDown and ResUp modules have the same architecture and calculation process. The main difference between them is that the ResDown module uses the 2D average pooling operation AvgPool2d, while the ResUp module uses the upsampling operation Upsample. In the ResDown module, the input passes through two computational paths to achieve the residual calculation. In one computational path, the input is sequentially processed through the 2D average pooling operation AvgPool2d and the 2D convolution operation Conv2d. In the other computational path, the input is first sequentially processed through the 2D convolution operation Conv2d and the 2D batch normalization operation BatchNorm2d, and then the calculation results are converted through the Randomized Leaky ReLU (RReLU) activation function. The calculation process of the RReLU activation function is as follows:

(8) $$RReLU\left(x\right)=\left\{\begin{array}{cc} x, & if\;x\geq 0\\ \lambda x, & otherwise \end{array}\right.$$

where λ∼U(1/8,1/3) denotes the scaled value randomly sampled from the uniform distribution *U*. The calculation results of the RReLU activation function are further processed sequentially through the 2D average pooling operation AvgPool2d, the 2D convolution operation Conv2d and the 2D batch normalization operation BatchNorm2d. The calculation results of the two computational paths are concatenated (“+” in Figure 9b) and then calculated through the RReLU activation function to obtain the output.

Compared to the AE-MLP model, the AE-RES model has a more complex architecture. However, the AE-RES model can effectively extract the category features of heterogeneous projection images even after one iteration. Therefore, we adopt the one-pass learning mode [52,53] to train the AE-RES model.

## 4. Conclusions

Heterogeneous projection image classification is an effective way for solving the structural heterogeneity problem of flexible biological macromolecules in single-particle cryo-EM. In this paper, we propose an unsupervised classification algorithm for heterogeneous cryo-EM projection images based on deep autoencoder models, which does not require any a priori knowledge such as the orientations or common lines of the heterogeneous projection images. We use multi-layer perceptrons and residual networks to implement the autoencoder model for extracting the category features of heterogeneous projection images. The multi-layer perceptron-based autoencoder is very fast to train, while the residual network-based autoencoder can extract the desired features in the one-pass learning mode. Image downsampling can reduce computational load and noise, while increasing the number of channels of images can enhance their semantic information. Applying the UMAP algorithm to reduce the high-dimensional latent variables to 2D can further aggregate the most important features, and applying the spectral clustering algorithm to cluster the 2D features can achieve higher classification accuracy. The classification performance of the proposed algorithm is demonstrated on a discrete compositional heterogeneous conformation dataset and a conformational heterogeneous dataset. Experimental results show that the proposed algorithm can accurately classify the projection images of discrete compositional conformations and also has advantages on the dataset of projection images of different conformations of a protein. In future work, we will further explore deep generative network models with more powerful learning and representation capabilities, and apply them to heterogeneous 3D reconstructions on real cryo-EM projection image datasets.

## Figures and Tables

**Table 1:** The main parameters for training the proposed AE-MLP and AE-RES models.

Parameters	AE-MLP	AE-RES
Dimensionality of Latent Variables	8	4096
Maximum Learning Epoch	500	1
Batch Size	32	32
Optimizer	SGD	SGD
Learning Rate	0.001	0.001

**Table 2:** The classification accuracy on the discrete compositional conformation cryo-EM dataset using the 2D features acquired from the AE-MLP model, the AE-RES model, and the original heterogeneous projection images.

Methods	EMD-0230	EMD-0230	EMD-0230	Mean
AE-MLP	100.00%	100.00%	100.00%	100.00%
AE-RES	100.00%	100.00%	100.00%	100.00%
Original Images	93.40%	100.00%	94.60%	96.00%

**Table 3:** The classification accuracy on the conformations of the bacterial ribosome cryo-EM dataset using the 2D features acquired from the AE-MLP model, the AE-RES model, and the original heterogeneous projection images.

Methods	EMD-8440	EMD-8441	EMD-8445	EMD-8450	Mean
AE-MLP	75.00%	60.40%	34.80%	60.20%	57.60%
AE-RES	98.00%	53.20%	37.40%	80.40%	67.25%
Original Images	30.80%	26.00%	21.80%	29.00%	26.90%

**Table 4:** The number of projection images grouped into different conformations of the bacterial ribosome using the 2D features acquired from the AE-MLP model, the AE-RES model, and the original heterogeneous projection images.

Methods	EMD-8440	EMD-8441	EMD-8445	EMD-8450
Ground Truth	500	500	500	500
AE-MLP	478	676	401	445
AE-RES	569	499	462	470
Original Images	580	507	406	507

## Data Availability

The code that supports this study is available from the corresponding author upon reasonable request. Publicly available datasets were used in this study. The published cryo-EM density maps can be downloaded from http://www.emdataresource.org/ (accessed on 8 March 2023).
